# Semi-automatic measurements of foot morphological parameters from 3D plantar foot scans

**DOI:** 10.1186/s13047-021-00461-z

**Published:** 2021-03-17

**Authors:** Giulia Rogati, Alberto Leardini, Maurizio Ortolani, Paolo Caravaggi

**Affiliations:** Movement Analysis Laboratory, IRCCS Istituto Ortopedico Rizzoli, Via di Barbiano 1/10, 40136 Bologna, Italy

**Keywords:** Foot, 3D scans, Custom software, Medial longitudinal arch, Foot morphological parameters

## Abstract

**Background:**

Foot healthcare research is focusing increasingly on personalized orthotic and prosthetic devices to address patient-specific morphology and ailments. Customization requires advanced 3D image processing tools to assess foot and leg geometrical parameters and alterations. The aim of this study is to present a new software for the measurement of the foot shape from 3D scans of the foot plantar surface.

**Methods:**

A Kinect-based scanning device was used to acquire the 3D foot shape of 44 healthy subjects. A software was developed in Matlab to measure the foot main morphological parameters from foot scans. Principal Component Analysis was used to orientate the foot scans with respect to the same reference system. Accuracy, via percentage errors and Bland-Altman plots, and correlation of the software-based foot parameters were assessed against manual measurements. A normalized Arch Volume Index (nAVI) was proposed and correlated to the traditional Arch Index. Test-retest Intraclass Correlation Coefficient was used to assess the inter-session repeatability of foot measurements.

**Results:**

The average percentage error between software and manual measurements was 1.2 ± 0.8% for foot length, 9.1 ± 3.7% for foot width, 22.3 ± 13.5% for arch height and 23.1 ± 12.7% for arch depth. Very strong correlations were observed for foot length (*R* = 0.97) and foot width (*R* = 0.83), and strong correlations for arch height (*R* = 0.62) and arch depth (*R* = 0.74). nAVI was negatively correlated to the Arch Index (*R* = -0.54). A small difference was found between software and manual measurements of foot length (Δ = 0.92 mm), a software overestimation of foot width (Δ = 8.6 mm) and underestimation of arch height (Δ = -1.4%) and arch depth (Δ = -11%). Moderate to excellent repeatability was observed for all measurements (0.67–0.99).

**Conclusions:**

The present software appears capable to estimate the foot main morphological parameters without the need for skin markers or for identification of anatomical landmarks. Moreover, measurements are not affected by the foot orientation on the scanning device. The good accuracy and repeatability of measurements make the software a potentially useful operator-independent tool for the assessment of foot morphological alterations and for orthotics customization. nAVI may be used for a more realistic classification of foot types when 3D foot images are available.

## Background

The foot, one of the most complex musculoskeletal structures in the human body, provides a stable support to the body and features variable compliance to address the varying dynamic conditions characterizing motor activities. A correct foot posture is fundamental to avoid the onset of chronic pain [1–3], lower limb injury or misalignment [4, 5] and balance-related issues [6]. Foot healthcare research is increasingly focusing on orthotic and prosthetic devices personalized on both patients morphology and functional demand [7–9]. In particular, additive manufacturing technology allows to obtain custom devices and complex-shaped prototypes from 3D scans of anatomical segments [10]. Although optical- and laser-based scanners are emerging as the new gold standard for the non-invasive acquisition of the 3D shape of leg and foot, the lack of automatic 3D processing tools and the initial outlay required for such technology have limited their wide application and diffusion; therefore traditional techniques are still in use [11]. However, foot shape acquisition via plaster cast and foam impression is time consuming and largely operator-dependent [12], and do not provide measurements of the foot shape [13]. In order to overcome traditional operator-dependent methods and to improve accuracy and repeatability of the anatomical replica, novel low-cost 3D scanning devices, suitable for clinical applications, have been developed and tested [14–16]. 3D scanning of the lower limb allows the design of custom devices, such as orthotic insoles, Ankle-Foot-Orthoses, special footwear and prosthetic limbs, and the measurement of foot and leg geometrical features. While callipers and measuring tapes have been the standard foot measuring systems to date [17, 18], the need for a faster, more comprehensive and objective data collection has stimulated the development of software for automatic measurements of the foot main morphological parameters from 3D scans [19, 20]. The foot is a rather complex anatomical structure, and some morphological features can be difficult to measure. While specific setups have been proposed [21, 22], very few automatic measurement systems capable to accurately measure foot morphology from 3D scans have been reported. Most of these systems require the positioning of anatomical landmarks on the foot [17, 23, 24], or their manual identification on 3D scans [25, 26]. Therefore, while 3D scanning technology is replacing physical casts with digital replica, foot measurements are still operator-dependent.

A software capable of extracting the main geometrical parameters from 3D foot scans could provide objective operator-independent data which may be used by podiatrists and clinicians in the assessment of morphological alterations, and for customization of footwear and orthotic devices. In foot biomechanics, this tool could be exploited to shed more light on the relationship between foot morphology and joint mechanics, in relation to different foot types or pathologies.

The aim of this study is to present a new software for the automatic measurement of the foot main geometrical parameters from 3D scans of the foot plantar surface. The software does not require skin markers, manual identification of anatomical landmarks and measurements are not affected by the orientation of the foot on the scanning plate.

## Methods

**Fig. 1:**
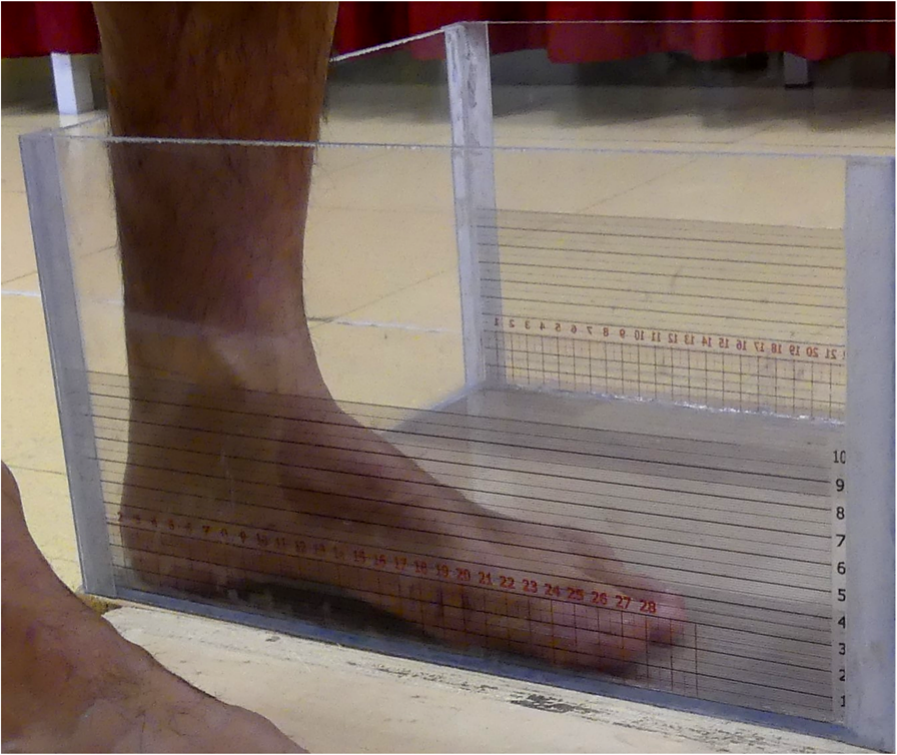
The PodoBox measurement tool. The plexiglass box used to measure the foot main morphological parameters. Detail of the adhesive rulers on the side walls for arch height and arch length measurement

**Fig. 2:**
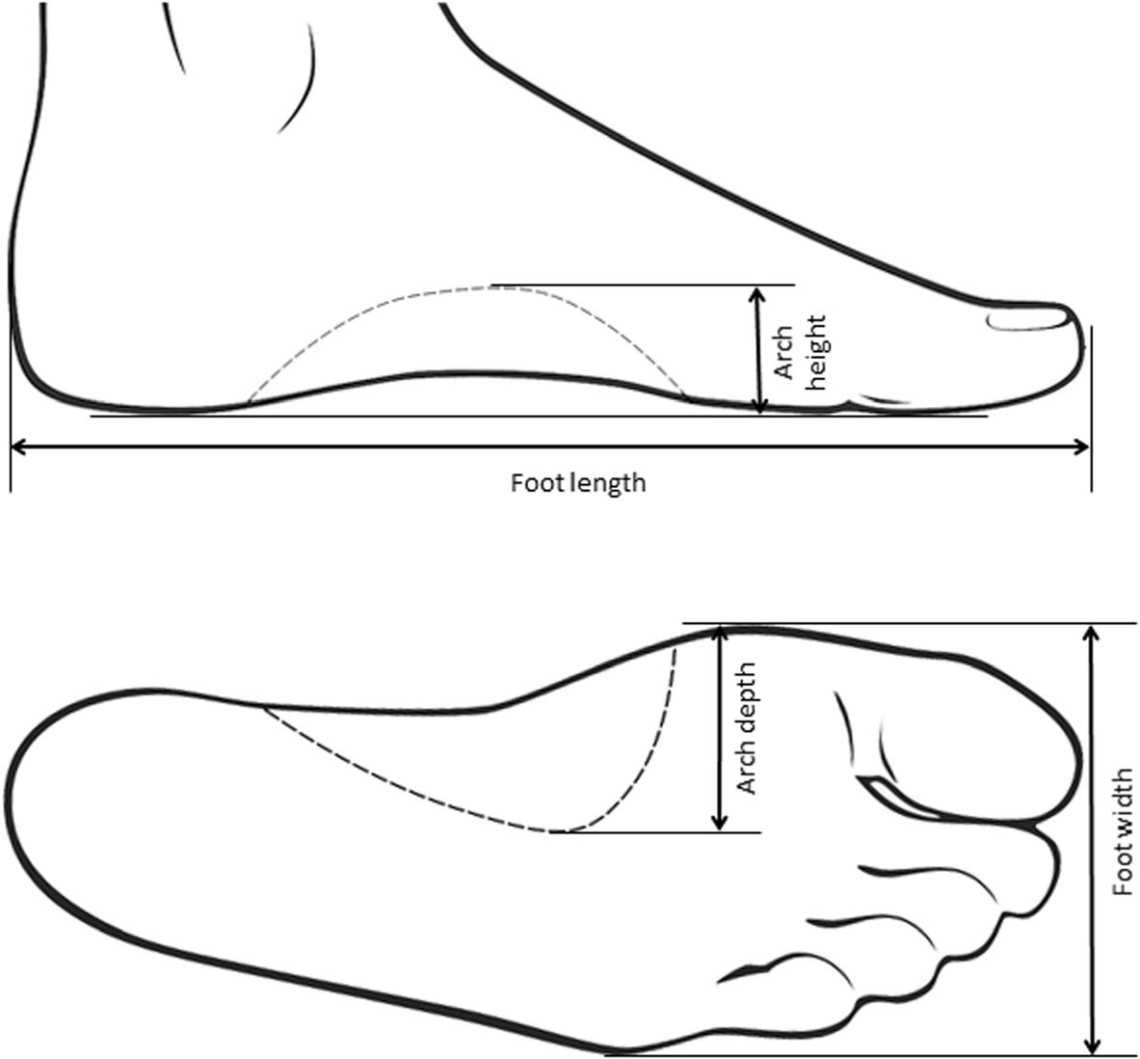
Foot morphological parameters. Top, foot length and arch height; bottom, foot width and arch depth

Forty-four healthy subjects (21 males & 23 females; age 20–63 years; BMI 22.0 ± 2.8 kg/m^2^, shoe size 36 – 45 EU) without foot or ankle pathologies and capable to ambulate independently volunteered for the study. Informed consent was obtained from all subjects after extensive explanation of the study aims and analyses involved. Subjects with healthy high-, normal- and low-arched feet were recruited as to include the major foot types in the population. Relevant foot anatomical landmarks were manually marked and a plexiglass measurement box (hereinafter called PodoBox, see [14]) fitted with rulers on the side walls and on the bottom surface (Fig. 1) was used to measure foot length, foot width, arch height and arch depth (maximum extension of the arch in the frontal plane) (Fig. 2). A validated Kinect-based [27] 3D foot scanner was used to acquire the plantar aspect of the feet of each volunteer in bipedal up-right standing posture [14]. The laptop computer used in the study (Intel Core i5 6300 HQ @2.30 GHz, 12Gb RAM) allowed to process each scan in few seconds. The software Skanect (Occipital, v1.8) was used to obtain a 3D mesh of the feet from the Kinect depth-data and allowed semi-automatic pre-processing of the image by removing noise and undesired parts. A finer editing was manually performed with Geomagic Control™ (3D Systems, Rock Hill, USA) to slightly smooth the foot plantar surface. The Kinect-based foot scanner and the protocol for image acquisition and editing was validated in a previous study by the same authors [14].

**Fig. 3:**
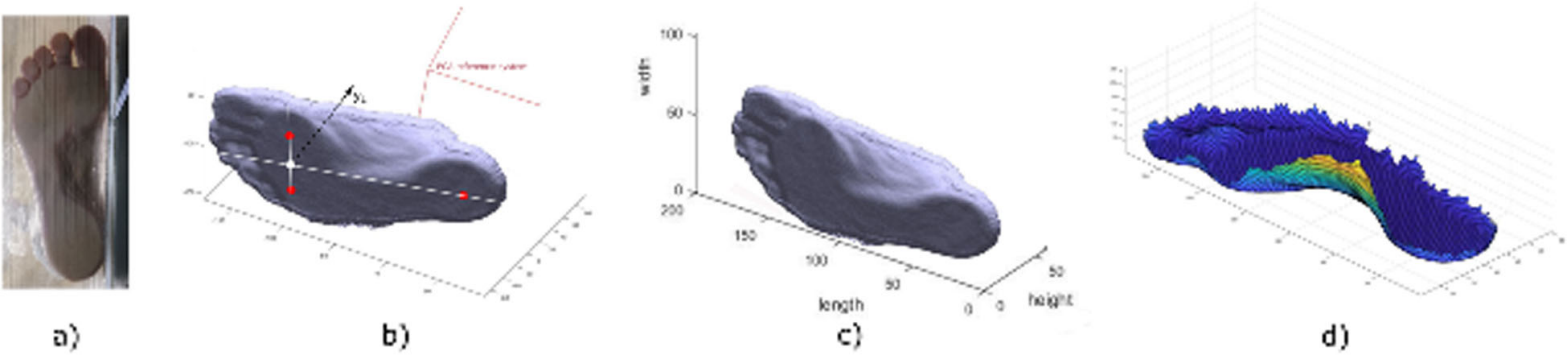
Software analysis of the 3D foot scans. **a** Acquisition of the foot plantar surface; **b** identification of three non-aligned surface points to find the three axes of the local reference frame; **c** alignment of the foot local reference frame with the global reference system; **d** 3D mapping of vertical position of foot surface on the I x J matrix, after smoothing and filtering

**Fig. 4:**
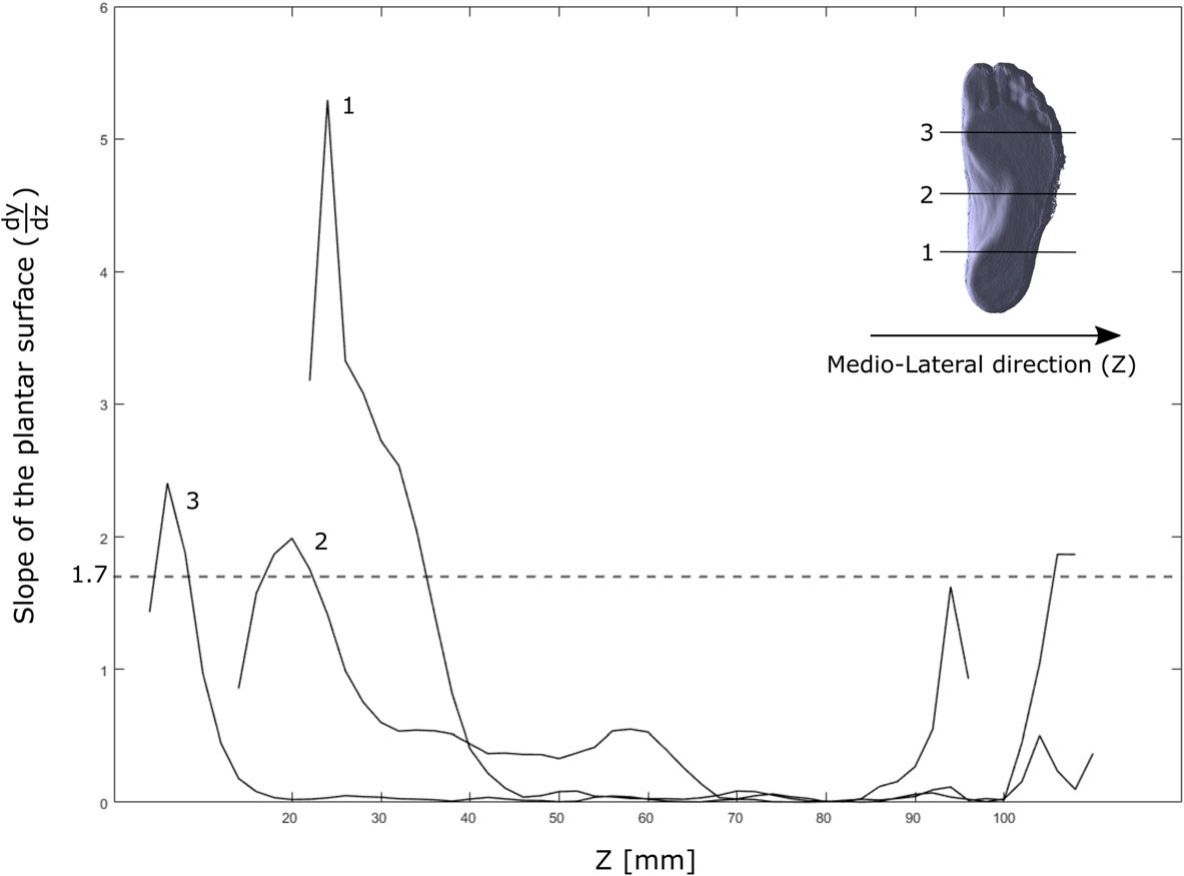
First derivative analysis of the plantar foot surface. For one foot of one subject, slope of the plantar surface in three planes parallel to the YZ plane. A threshold of the first derivative dy/dz = 1.7 (i.e. 60 deg slope) was used to identify and separate the plantar surface region from the foot sides

**Fig. 5:**
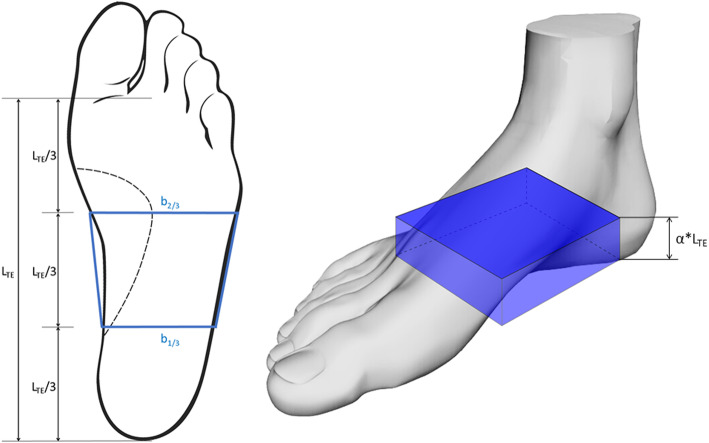
Reference volume for nAVI normalization. Left, the base of the reference volume, where b_1/3_ and b_2/3_ are the widths of the 3D foot scan at 1/3 and 2/3 of L_TE._ Right, the reference volume superimposed to the foot; the height of the reference volume is α*L_TE_ (see Eqs. 1 and 2)

A custom software to extract the foot main geometrical parameters from the foot scans was developed in Matlab (version r2016a, MathWorks) (Fig. 3). The 3D scans, as depth data, were saved as STL files and imported in Matlab as n × 3 matrices, where n is the number of nodes comprising the mesh. In order to align the 3D images with respect to a common reference system, Principal Component Analysis (PCA) was used to find the three principal directions of variance of the point cloud. These three orthogonal unit vectors were used for the preliminary rough alignment of the image with respect to the X (antero-posterior), Y (vertical) and Z (medio-lateral) axes of the global reference system. A local reference frame was defined on the 3D foot scan by automatic identification of three non-aligned points on the foot plantar surface: one at the centre of the rearfoot, and two approximately under the head of the first and fifth metatarsal bones. These points, based on geometrical parameters, allowed to define the longitudinal axis (X_L_) and the normal to the ground plane, assumed to be the vertical axis (Y_L_) of the local reference system (Fig. 3b). The medio-lateral axis (Z_L_) was obtained by the cross-product of the other two. A rotation matrix was applied to the point cloud to refine the 3D image alignment to the global reference system (Fig. 3c). Foot length is computed as the largest distance between two points along the X axis, whereas foot width as the largest distance along the Z axis. In order to measure the other morphological parameters, the 3D point cloud is mapped into a I x J matrix (M, with a resolution of 2 × 2 mm), where the element m_i,j_ stores the vertical position (along Y) of the plantar surface point located at coordinates i (along X) and j (along Z). A 2 × 2 moving average filter is applied to slightly smooth the surface and reduce possible irregularities due to the 2D mapping process. A threshold in the first derivative across columns ($\frac{dy}{dz}$) of matrix M was used to remove the parts of the foot surface presenting a slope in the YZ plane larger than 60 deg (Fig. 4). This angle was assumed to define the limit between foot surface and lateral side of the foot (Fig. 3d) and was identified following an optimization analysis using the manually-measured arch heights as target values. The analysis was conducted on 10 feet and the optimized values were used for the accuracy assessment performed on the remaining 78 feet. Arch height and arch depth were computed by identifying and isolating the plantar arch region - the points in the central third of the 3D surface with y > 0 - from the foot plantar surface in contact with the ground - the points with y ≈ 0. Arch height was defined as the highest point (along Y) of the arch region, and arch depth as the maximum width along Z. To allow inter-subject comparison, these measurements are reported and normalized to the foot length and foot width at midfoot (50% of foot length), respectively. The Arch Index (AI) [28], defined as the ratio between the area of the middle third of the footprint and the total area of the footprint toes excluded, was computed following identification of the points in contact with the ground. In order to extend the AI definition to the real 3D morphology of the foot arch [29], a normalized Arch Volume Index (nAVI) representing the volume covered by the arch of the foot – or arch volume – was defined and computed (Eq. 1). nAVI was expressed as the ratio of the arch volume to a reference volume comprised by a prism with trapezoidal base, and height defined as the length of the foot multiplied by a coefficient α = 0.122 (Eq. 2, Fig. 5).

1 $$ \mathrm{nAVI}=\frac{{\mathrm{V}}_{\mathrm{arch}}}{{\mathrm{V}}_{\mathrm{ref}}}\ast 100\ \left[\%\right] $$

2 $$ {V}_{ref}=\frac{\left({b}_{\raisebox{1ex}{$1$}\!\left/ \!\raisebox{-1ex}{$3$}\right.}+{b}_{\raisebox{1ex}{$2$}\!\left/ \!\raisebox{-1ex}{$3$}\right.}\right)}{2}\ast \frac{L_{TE}}{3}\ast 0.122\ast {L}_{TE}\approx 0.02\ast \left({b}_{\raisebox{1ex}{$1$}\!\left/ \!\raisebox{-1ex}{$3$}\right.}+{b}_{\raisebox{1ex}{$2$}\!\left/ \!\raisebox{-1ex}{$3$}\right.}\right)\ast {L_{TE}}^2\kern1.25em \left[{mm}^3\right] $$

Where b_1/3_ and b_2/3_ are the widths of the 3D foot shape along the Z axis at 1/3 and 2/3 of L_TE_ (foot length toes excluded), respectively (Fig. 5). α was estimated as the ratio between arch height and L_TE_ and averaged over 78 feet. Following this definition, nAVI of a fully flat foot would be ≈ 0, since the arch volume would be close to zero, and that of an extremely cavus foot, presenting a large arch height and thus a large arch volume, would be ≈ 1.

The accuracy of the custom software (% error) was evaluated by comparing the computed foot measurements to those using the PodoBox. Correlation between software and PodoBox measurements was assessed via Pearson’s coefficients and Bland-Altman plots [30, 31]. Test-retest Intraclass Correlation Coefficients ICC (3,1) was used to assess the repeatability of the software in measuring foot length, foot width, arch height, arch depth and AI in 10 subjects (20 feet), acquired in three sessions in the same weight-bearing conditions.

## Results

### Accuracy study

**Fig. 6:**
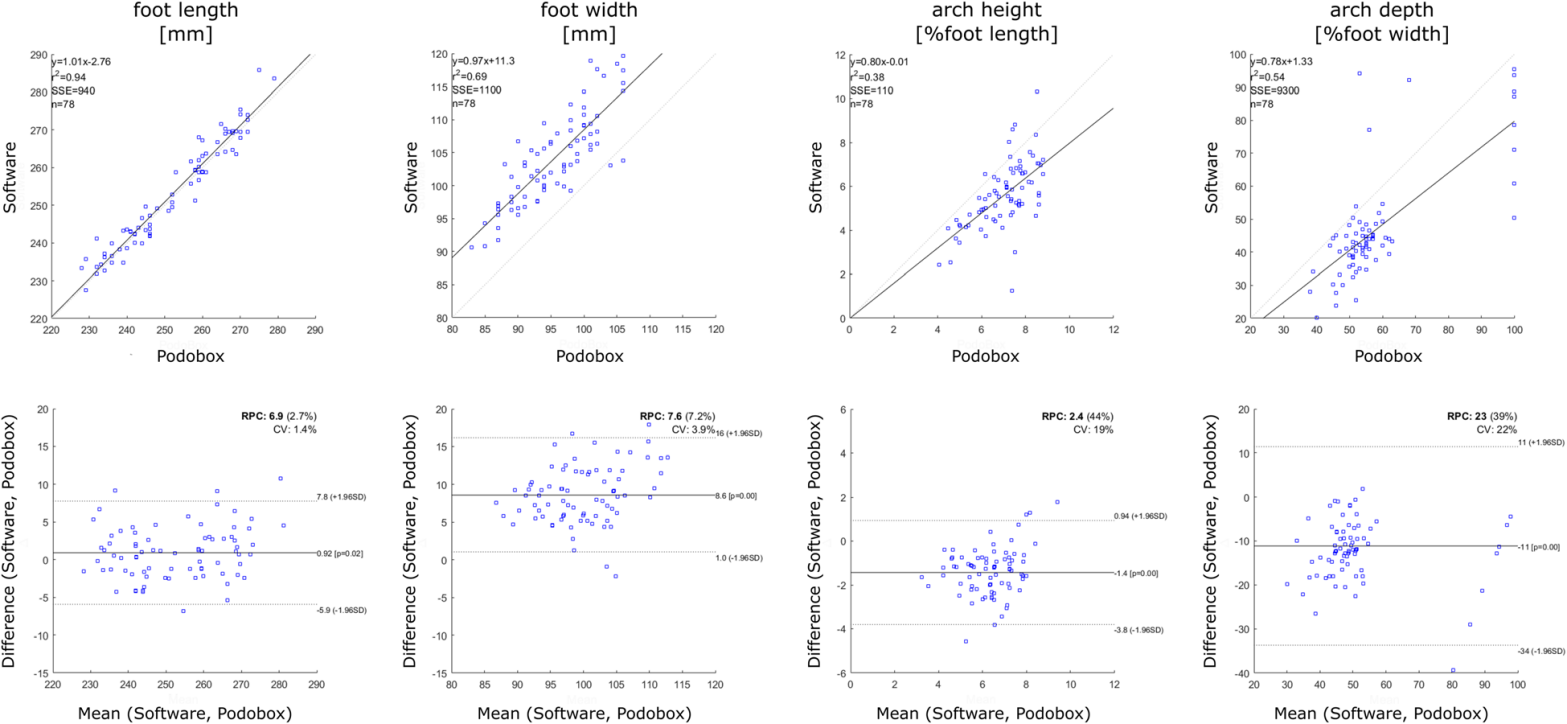
Correlation and Bland-Altman plots. (Top) Scatter plots of the linear correlations between PodoBox and software measurements of foot length, foot width, arch height and arch depth in 78 feet. (Bottom) Bland-Altman scatter plots showing the difference (Y axis) and the mean (X axis) between PodoBox and software measurements

Non-parametric paired Wilcoxon signed-rank test did not find any significant difference in PodoBox measurements between left and right foot across all participants (Table 1). The average percentage error between software and PodoBox measurements was 1.2 ± 0.8% for foot length, 9.1 ± 3.7% for foot width, 22.3 ± 13.5% for arch height and 23.1 ± 12.7% for arch depth. The linear correlation analysis (*p* < 0.01) showed very strong correlations between software and PodoBox measurements for both foot length (*R* = 0.97) and foot width (*R* = 0.83), and strong correlations for arch height (*R* = 0.62) and arch depth (*R* = 0.74) (Fig. 6, top). nAVI was found to be negatively correlated to AI (*R* = − 0.54, *p* < 0.01). A very small difference was found (Fig. 6, bottom) between PodoBox and software measurements of foot length (Δ = 0.92 mm), an overall software overestimation of foot width (Δ = 8.6 mm) and underestimation of arch height (Δ = − 1.4%) and arch depth (Δ = − 11.0%).

**Table 1:** PodoBox measurements. Median [25% 75%] foot geometrical parameters in the left and right feet of 44 participants assessed by the Podobox. Statistical differences between left and right measurements were assessed via Wilcoxon signed-rank test (α = 0.05)

	LEFT	RIGHT	p
**Foot length [mm]**	253 [242 265]	252 [242 265]	NS
**Foot width [mm]**	95 [92 100]	96 [91 100]	NS
**Arch height [mm]**	18 [16 20]	18 [15 20]	NS
**Arch height [%foot length]**	7.3 [6.6 7.9]	7.2 [6.0 7.8]	*p* = 0.056
**Arch depth [%]**	55 [51 57]	54 [50 56]	NS

### Repeatability study

Test-retest ICC (3,1) showed excellent repeatability for the software-based foot length and foot width measurements (0.99 and 0.93, respectively). Strong repeatability was observed for arch height (0.80) and arch depth (0.82), while moderate repeatability was found for AI (0.67).

## Discussion

There is a continuous and growing interest in custom devices and orthotics for foot care. Customization has been used to improve comfort of sport [32] and safety shoes orthotics [33], and to address patients’ needs via personalized orthoses to support or restore foot and ankle function [34]. In most cases, custom solutions are based on 3D models of the foot shape obtained via laser scanners or time-of-flight sensors technology [11, 35]. While laser scanners are still the gold standard to acquire foot morphology, low-cost scanning devices with adequate accuracy and usability for most applications are emerging [14].

In this study, we are showing accuracy and repeatability of a new software designed to estimate foot dimensions and other clinically-relevant anatomical features from 3D foot scans. All foot measurements showed strong or very strong correlation with the corresponding manual measurements thus proving the software consistency in detecting the correct anatomical features (Fig. 6, top). The software was capable of measuring foot length with high accuracy; foot width was slightly overestimated, as shown by the positive mean difference between software and PodoBox measurements (Fig. 6, bottom). This bias was most likely a consequence of the soft tissue compression in the medial and lateral side of the foot during PodoBox measurements. Due to their complex definition and to small differences in foot posture and arch shape between Podobox measurements and Kinect scanning, larger percentage errors were indeed expected for arch height and arch depth. However, a consistent negative bias was observed (Fig. 6, bottom), therefore a larger accuracy might be obtained by further improving the software calculations of these two measures.

nAVI was established to quantify the arch volume normalized to a reference volume. The nAVI of 88 feet showed significant negative correlation (*R* = − 0.54) with the software-computed traditional AI. As expected, a larger arch volume, which results in a larger nAVI, is associated to a lower midfoot-to-ground contact area - thus to a lower AI. Whenever the 3D shape of the foot is available, nAVI may be used to overcome the traditional classification of foot types based on the AI to improve our understanding of foot types. Furthermore, nAVI could be used to investigate foot flexibility, by analysing variation of the arch volume in different loading conditions.

The test-retest ICC showed excellent repeatability for foot length and foot width measurements, and moderate to good repeatability for arch height, arch depth and AI measurements. These results highlight the robustness of the software in computing foot length and foot width, as these measurements are the least affected by the foot position and loading conditions on the scanning device. The arch shape, conversely, may be more affected by the loading conditions; therefore, arch height, arch depth and AI measurements are expected to present larger variability across different sessions. These results further confirm the robustness of the measurements regardless of the foot orientation on the scanning plate.

The procedure is almost fully automatic, but the Kinect foot scans have required some manual editing. In addition, while the software computes the measurements on a “clean” 3D foot scan in less than 60 s, about 60 min are necessary for the whole process including the scanning. It should be emphasized that, despite the average quality of the present 3D scans acquired with an extremely low-cost scanning device [27], all measurements showed strong correlations with the corresponding manual measurements. Therefore, better performances are expected by employing higher-resolution 3D plantar foot scans. Since in current orthotic practice custom insoles are also designed from non weight-bearing foot scans, software accuracy should be assessed with respect to this loading condition. Finally, validation of the procedure would benefit from further assessment on a population of pathological feet requiring orthotics.

## Conclusions

This study presented a new software for the semi-automatic estimation of the foot main morphological parameters from 3D plantar foot scans, without the need for skin-markers or identification of anatomical landmarks. While the accuracy in the estimation of some parameters can be further improved, the software has the potential to become a useful operator-independent tool for the assessment of foot pathologies and major morphological alterations.

## Data Availability

The datasets used and/or analysed during the current study are available from the corresponding author on reasonable request.

## Abbreviations

3D: Three-Dimensional
BMI: Body Mass Index
AI: Arch Index
PCA: Principal Component Analysis
nAVI: Normalized Arch Volume Index
ICC: Intraclass Correlation Coefficient
